# Effects of a 10-week need-supportive teaching program on motor competence in children ages 7–10 with motor difficulties: a single-group repeated-measures design

**DOI:** 10.3389/fpsyg.2026.1909241

**Published:** 2026-09-24

**Authors:** TianYue Zhao, YiYao Peng, ZiXin Chen, Xiaojun Wang

**Affiliations:** 1 School of Physical Education, Sichuan University, Chengdu, Sichuan, China; 2 School of Law, Sichuan University, Chengdu, Sichuan, China

**Keywords:** developmental coordination disorder, fundamental movement skills, motor competence, motor difficulties, need-supportive teaching

## Abstract

**Background:**

Children with motor difficulties potentially associated with developmental coordination disorder (DCD) often face challenges in school-based physical activity settings, leading to reduced perceived competence, frustration, and avoidance of movement tasks. Need-supportive teaching, grounded in self-determination theory, emphasizes autonomy, competence, and relatedness support within structured learning environments and may provide a psychologically supportive context for motor learning.

**Objective:**

This study aimed to examine changes in motor competence during a 10-week need-supportive teaching intervention among children ages 7–10 years with motor difficulties identified through school-based screening.

**Methods:**

A single-group repeated-measures design was used. Twenty children identified via questionnaire screening for motor coordination difficulties with an MABC-2 total percentile score at or below the 16th percentile (13 boys and 7 girls; mean age, 8.38 ± 1.00 years) participated in a 10-week school-based need-supportive teaching program delivered three times per week, 40 min per session. The program incorporated meaningful choices, progressively graded motor tasks, specific process-focused feedback, and cooperative activities. Outcomes included the MABC-2 total, Manual Dexterity, Aiming and Catching, and Balance standard scores assessed at baseline (T0), after Week 5 (T1), and immediately after Week 10 (T2).

**Results:**

The median (Q1, Q3) MABC-2 total standard score was 5.50 (5.00, 6.25) at T0, 6.00 (5.00, 7.00) at T1, and 7.00 (6.00, 8.00) at T2. The Friedman test indicated significant changes in performance across the time points, *χ*^2^(2) = 27.825, *p* < 0.001, Kendall’s *W* = 0.696. Holm-adjusted Wilcoxon signed-rank tests showed no significant difference between T0 and T1 (adjusted *p* = 0.132), but a significant improvement between T0 and T2 (adjusted *p* < 0.001). Significant performance changes also occurred across the time points for Manual Dexterity (*χ*^2^(2) = 20.320, *p* < 0.001), Aiming and Catching (*χ*^2^(2) = 25.033, *p* < 0.001), and Balance (*χ*^2^(2) = 14.966, *p* < 0.001). Aiming and Catching showed the largest median increase from T0 to T2.

**Conclusion:**

Motor competence improved over the 10-week program, particularly from T1 to T2, with the largest median increase observed in Aiming and Catching. These preliminary findings should be interpreted cautiously given the single-group design and possible task-specific practice effects.

## 1 Introduction

Developmental Coordination Disorder (DCD) is a common neurodevelopmental condition affecting approximately 5–6% of school-aged children. It is characterized by difficulties acquiring and executing coordinated motor skills that fall below age expectations and cannot be better explained by intellectual disability, visual impairment, or other neurological conditions (American Psychological Association, 2013; Blank et al., 2019; World Health Organization, 2019). These motor difficulties may interfere with daily living, academic productivity, physical education, play, and social participation (Missiuna et al., 2007; Zwicker et al., 2012; Van der Linde et al., 2015; Wilson et al., 2017). Children with DCD often show clumsiness, slowness, poor accuracy, and difficulty learning age-appropriate motor tasks, and these problems may persist without adequate support (Cantell et al., 2003; Kirby et al., 2008; Blank et al., 2019). They are also more likely to experience reduced physical activity, poorer fitness, lower perceived motor competence, and psychosocial difficulties, including frustration, avoidance, peer-related stress, and emotional distress in movement-related contexts (Rivilis et al., 2011; Cairney et al., 2013; Missiuna et al., 2014; Li et al., 2018).

Motor difficulties in DCD are heterogeneous and may involve fine motor control, object control, postural stability, motor planning, and sensorimotor integration (Zwicker et al., 2009; Wilson et al., 2013; Wilson et al., 2017; Biotteau et al., 2019). Some children mainly experience handwriting or manual dexterity difficulties, while others have greater problems in ball skills, balance, or whole-body coordination (Smits-Engelsman et al., 2001; Prunty et al., 2016; Barnett and Prunty, 2021). Motor competence in children with DCD or motor coordination difficulties is commonly assessed using the Movement Assessment Battery for Children-Second Edition (MABC-2), which includes Manual Dexterity, Aiming and Catching, and Balance (Henderson et al., 2007; Schulz et al., 2011; Blank et al., 2019). Because impairment may differ across these domains, intervention studies should report total, domain-specific, and task-specific patterns of change (Schulz et al., 2011; Wuang et al., 2012; Wilson et al., 2017).

Evidence for DCD intervention increasingly supports motor-based, task-oriented, and activity-oriented approaches. Recent reviews continue to show that DCD interventions are predominantly organized around task-oriented motor practice, motor-skill or sports training, process-oriented approaches, and technology-assisted training, whereas longer-term psychosocial and participation outcomes remain comparatively underexplored (Gao et al., 2025; Jelsma et al., 2026). Reviews and meta-analyses suggest that structured practice, meaningful tasks, progressive challenges, and repeated skill execution can improve motor performance, with task-oriented approaches showing stronger effects than process-oriented approaches alone (Wilson, 2005; Hillier, 2007; Smits-Engelsman et al., 2013; Miyahara et al., 2017). Virtual reality, Wii-based or exergame-based, balance-oriented, and activity-based interventions have also shown benefits for standardized motor outcomes or specific motor tasks (Wuang et al., 2011; Ferguson et al., 2013; Purpura et al., 2024). However, effects may vary across domains and may not generalize equally to participation, psychosocial outcomes, or sustained physical activity engagement (Hillier, 2007; Smits-Engelsman et al., 2013; Miyahara et al., 2017). Because children with DCD often experience motor failure, embarrassment, social comparison, and avoidance, interventions should consider not only motor practice but also the pedagogical and motivational climate in which it occurs (Cairney et al., 2013; Missiuna et al., 2014; Zimmer et al., 2020).

From a psychological perspective, children’s motor coordination difficulties are not only performance-related problems but may also shape motivational experiences in physical activity settings. Repeated failure, peer comparison, and uncertainty about task success may reduce perceived competence and increase avoidance of movement tasks. Therefore, a supportive learning environment may help children attempt challenging tasks, persist after errors, and participate more actively in repeated motor practice. This perspective links school-based motor intervention with psychological theories of motivation and engagement. It provides a rationale for examining need-supportive teaching as a pedagogical framework for children with motor coordination difficulties.

Need-supportive teaching offers a theoretically grounded approach based on Self-Determination Theory, which emphasizes support for autonomy, competence, and relatedness (Deci and Ryan, 2000; Ryan and Deci, 2000; Vansteenkiste et al., 2020). In physical education, autonomy support involves meaningful choice and acknowledgment of children’s perspectives; competence support involves clear structure, achievable challenges, demonstrations, progressive difficulty, and specific feedback; and relatedness support involves a warm, respectful, and cooperative climate (Reeve, 2006; Cox and Williams, 2008; Aelterman et al., 2014; Haerens et al., 2015). Previous research has linked need-supportive or autonomy-supportive teaching with adaptive motivation (i.e., more autonomous forms of motivation characterized by interest, personal value, and volitional engagement), psychological need satisfaction, engagement, and positive learning experiences in physical education (Standage et al., 2005; Cox and Williams, 2008; Chatzisarantis and Hagger, 2009; Cheon et al., 2012; Haerens et al., 2015; Aelterman et al., 2019). More specifically, Vlachopoulos et al. (2021) examined self-determined motivation for physical activity among adolescents with and without DCD in school physical education, highlighting the relevance of motivational regulation in this population and the importance of supportive physical education environments for students with motor difficulties. Evidence from school-based physical activity and motor-skill contexts further suggests that these effects may extend beyond motivational outcomes, although findings are not uniformly positive. Chan et al. (2019), in a cluster-randomized trial involving 282 primary schoolchildren, reported significant intervention effects on locomotor skills (adjusted mean difference = 2.47, Cohen’s *d* = 0.76) and overall fundamental movement skills (adjusted mean difference = 3.72, *d* = 0.93), whereas changes in perceived competence and enjoyment were less evident. Lee et al. (2020) examined an 8-week, 24-session need-supportive motor-skill intervention. They found significant group effects for locomotor competence (partial *η*^2^ = 0.11), object-control competence (partial *η*^2^ = 0.24), and school-time moderate-to-vigorous physical activity (partial *η*^2^ = 0.19). In inclusive physical education, Behzadnia et al. (2022) reported that students with mild or borderline intellectual disability taught by need-supportive teachers experienced greater psychological need satisfaction and positive affect and lower need frustration, amotivation, and negative affect than students receiving usual teaching. Conversely, Sebire et al. (2016), in an SDT-based extracurricular physical activity program, reported mixed motivational effects and only moderate theoretical fidelity, highlighting that the intended benefits of need-supportive teaching may depend on how consistently its principles are implemented. Importantly, need-supportive teaching is not defined by motor tasks that are fundamentally different from those used in conventional motor-skills training; rather, its intended distinction lies in how practice is organized and delivered through meaningful choice, individually adjusted challenge, specific informational feedback, supportive interpersonal interactions, and clear instructional structure.

The OPTIMAL (Optimizing Performance Through Intrinsic Motivation and Attention for Learning) framework offers a complementary perspective on the theory of motor learning, which proposes that autonomy support, enhanced expectancies, and an external focus of attention can facilitate motor performance and learning (Wulf and Lewthwaite, 2016). This framework partially overlaps with Self-Determination Theory, particularly in its emphasis on autonomy-supportive learning conditions and positive competence-related experiences, but it places greater emphasis on attentional processes directly related to motor learning. In children, Abdollahipour et al. (2017) found that providing a small choice to support autonomy and directing attention externally produced additive benefits for performance on a bowling task. More directly relevant to children with motor coordination difficulties, Shahbaz et al. (2024) examined children aged 7–10 years identified by the authors as being at risk for DCD and reported that combining self-controlled practice with a visual-illusion manipulation intended to enhance success expectancies resulted in better golf-putting performance in the retention test than in one comparison condition. However, comparable effects were not observed during practice or in the transfer test. These findings suggest that motivational and attentional conditions surrounding motor practice may matter for motor learning in children, including those with motor coordination difficulties. However, the present intervention was primarily grounded in Self-Determination Theory and did not systematically manipulate external focus of attention; therefore, it should not be considered a full OPTIMAL-theory intervention.

Despite these related findings, evidence on applying need-supportive pedagogical approaches to children with DCD or motor coordination difficulties remains limited. Recent reviews provide a clearer picture of the dominant intervention approaches in this population. Gao et al. (2025) found that motor-based randomized trials were primarily task-oriented, process-oriented, or a combination of both, with task-oriented interventions representing the largest category and producing a significant moderate effect on overall motor skills. More recently, Jelsma et al. (2026) identified 206 interventions across 202 DCD studies and found that task-oriented training, motor-skill and sports training, active video games, and sensory-perceptual motor approaches were among the most frequently used intervention types, while long-term psychosocial and participation outcomes were examined less often. Together with recent virtual-reality and task-specific motor-learning studies (Purpura et al., 2024; Shahbaz et al., 2024), this evidence indicates that the DCD intervention literature has predominantly targeted motor performance and task learning rather than sustained school-based pedagogical climates grounded in psychological need support. Comparatively less is known about whether need-supportive principles can be embedded across a sustained school-based motor program spanning multiple motor domains.

Accordingly, this study aimed to examine changes in the MABC-2 total and domain standard scores at baseline (T0), after Week 5 (T1), and immediately after Week 10 (T2) among children aged 7–10 years with motor difficulties participating in a 10-week need-supportive teaching program. Given the single-group repeated-measures design, the study was intended to characterize within-participant changes over time rather than establish causal efficacy. We hypothesized that the MABC-2 total standard score would improve across the three time points. We further hypothesized positive changes in Manual Dexterity, Aiming and Catching, and Balance, although the magnitude and timing of change might differ across domains. Task-specific analyses were exploratory, and no directional hypotheses were specified for individual tasks.

## 2 Method

### 2.1 Participants

Participants were children aged 7–10 years recruited from Grades 1 to 3 at a primary school in Chengdu, China. A two-stage screening procedure was used to identify children with motor coordination difficulties. First, 330 students were invited to complete a questionnaire-based screening for possible motor coordination difficulties. Of these, 209 children did not meet the screening criteria, and 121 children were referred for further motor assessment. This step served as a pragmatic school-based screening procedure and was not intended to provide a clinical diagnosis.

The 121 children who passed questionnaire screening were then assessed using the Movement Assessment Battery for Children-Second Edition (MABC-2). Children whose MABC-2 total percentile scores were above the 16th percentile were excluded. The remaining 20 children, who scored at or below the 16th percentile, were enrolled in the intervention. The recruitment and screening process is shown in Figure 1. Because classification was based on questionnaire screening and MABC-2 performance rather than a full DSM-5 diagnostic procedure, participants were described as children with motor difficulties identified through school-based screening rather than as children with clinically confirmed DCD. The MABC-2 was used as a standardized measure of motor performance and as one component of the study’s school-based identification procedure; it was not treated as a stand-alone clinical diagnostic instrument. Accordingly, the study used an MABC-2 total percentile score at or below the 16th percentile as the study’s motor-performance threshold for identifying children with motor difficulties, rather than as confirmation of a clinical diagnosis of DCD. The final sample included 13 boys (65.0%) and 7 girls (35.0%), with a mean age of 8.38 ± 1.00 years and a baseline MABC-2 total standard score of 5.45 ± 1.47.

**Figure 1:**
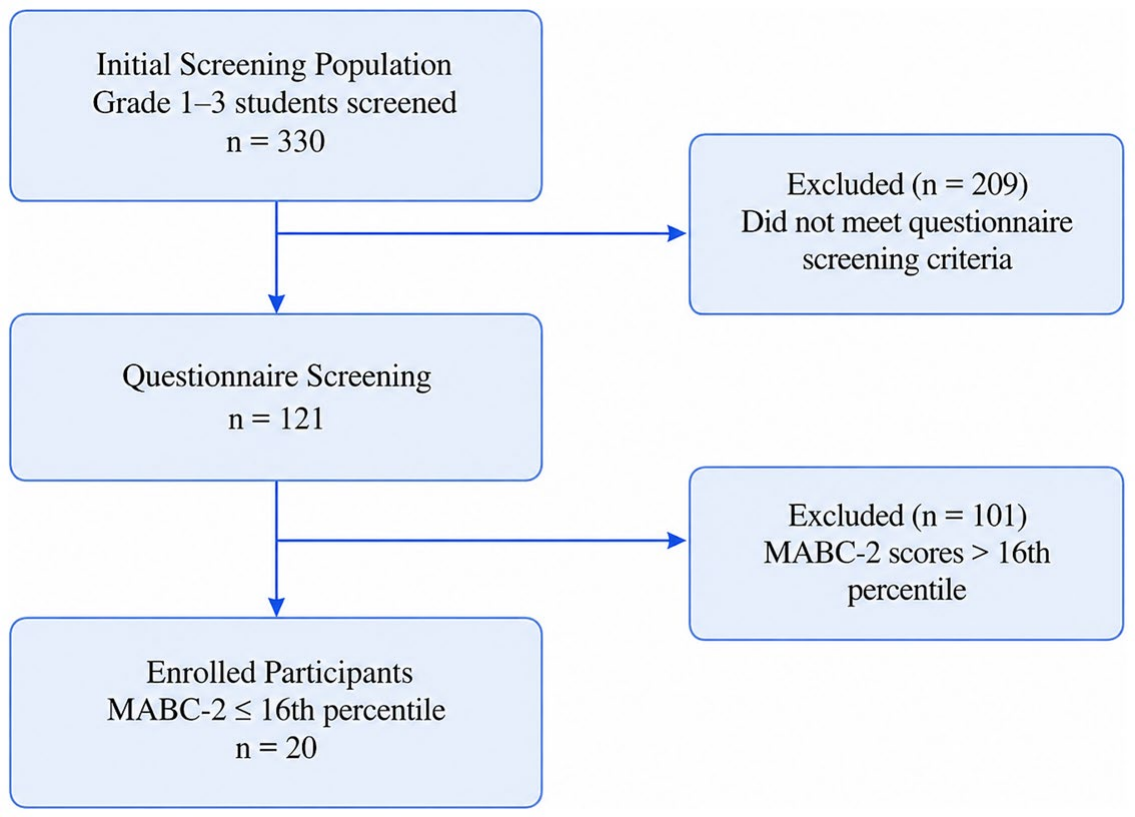
Flow diagram of participant screening and enrollment. MABC-2, Movement Assessment Battery for Children, Second Edition.

Inclusion criteria:

1. MABC-2 total percentile score at or below the 16th percentile;
2. Age between 7 and 10 years, corresponding to MABC-2 Age Band 2;
3. Meeting the questionnaire screening criteria for possible motor coordination difficulties;
4. Ability to understand and follow basic teaching instructions;
5. No systematic motor rehabilitation or specialized sports training within the previous 3 months;
6. Written informed consent provided by a parent or legal guardian, together with child assent;
7. Ability to participate in the 10-week intervention and complete all three assessments.

Exclusion criteria:

1. Diagnosed neurological or neuromuscular disorders clearly affecting motor function;
2. Severe visual or hearing impairment affecting test or intervention participation;
3. Obvious intellectual disability preventing understanding of basic instructions;
4. A documented diagnosis of ADHD or another neurodevelopmental condition affecting participation or assessment.
5. Acute injury or medical conditions contraindicating physical activity;
6. Withdrawal of guardian consent during the study;
7. Inability to complete the intervention or the repeated assessments.

Children with a documented diagnosis of ADHD or another relevant neurodevelopmental condition were excluded based on information available during participant screening. However, no standardized ADHD diagnostic assessment was administered as part of the study; therefore, previously undiagnosed comorbid ADHD could not be completely ruled out. Accordingly, this cohort should be regarded as a school-based sample of children with motor difficulties identified through questionnaire screening and standardized MABC-2 motor assessment rather than as a sample of children with a confirmed clinical diagnosis of DCD.

Sample size calculation.

An *a priori* sample size calculation was conducted using G*Power version 3.1.9.7 (Heinrich-Heine-Universität Düsseldorf, Düsseldorf, Germany) (Faul et al., 2007, 2009; Kang, 2021). For a one-group, three-time-point repeated-measures design, assuming an effect size of *f* = 0.30, α = 0.05, power = 0.80, a correlation of 0.50 among repeated measures, and a nonsphericity correction of 1.0, the minimum required sample size was 20 participants. The repeated-measures ANOVA framework was used as a practical approximation for sample size planning, whereas the final analyses used non-parametric tests because most change scores did not satisfy the normality assumption.

### 2.2 Study design and need-supportive teaching intervention protocol

This single-group repeated-measures study evaluated within-participant changes during a 10-week need-supportive teaching program comprising 30 school-based sessions delivered three times per week for 40 min per session. The study was conducted in Chengdu, China, between March 1 and May 4, 2026. Trained physical education teachers or researchers delivered the program using a standardized teaching manual in a physical activity room, playground, or multifunctional activity space. The study procedures were approved by the Medical Ethics Committee of Sichuan University (Approval No. K2025007) and followed the Declaration of Helsinki. Written guardian consent and child assent were obtained before enrollment. Where feasible, trained assessors who were not involved in program delivery conducted the outcome assessments; participants and instructors could not be blinded because of the educational nature of the program.

Each session followed a fixed structure: 5 min of warm-up activities, 20 min of fundamental movement skill practice, 10 min of game-based integrated activities, and 5 min of cool-down and feedback. The intervention content was aligned with the three MABC-2 motor domains: Manual Dexterity, Aiming and Catching, and Balance. Manual dexterity activities included small-object manipulation, bead-threading games, clipping tasks, coin-posting games, and tracing or maze-based activities. Aiming and catching activities included beanbag catching, rolling and catching balls, target throwing, and throwing light balls of different sizes. Balance activities included single-leg standing games, toe-walking tasks, grid jumping, balance beam walking, and obstacle-crossing activities. These activities were delivered as playful motor practice rather than as direct rehearsal of MABC-2 scoring procedures. Nevertheless, several intervention activities were similar to the motor demands assessed by the MABC-2, so that subsequent score changes may have reflected both broader motor learning and task-specific learning.

To enhance reproducibility, the intervention was manualized with predefined session structures, weekly objectives, task content, progressive task levels, and examples of teacher feedback and need-supportive teaching strategies. Need-supportive teaching was operationalized through four components. Autonomy support was provided through limited but meaningful choices, such as equipment type or color, task order, partner selection, or difficulty level. Competence support was provided through progressive task difficulty, demonstrations, specific feedback, and experiences of achievable success. Relatedness support included teacher encouragement, attention to children’s emotional responses, peer cooperation, and a supportive group climate. Structure support included stable routines, clear goals, simple rules, predictable activity sequences, and consistent safety expectations.

The distinction between motor-practice content and need-supportive delivery is described below. The intervention was not intended to differ from conventional motor-skills training primarily in the motor tasks practiced. Activities targeting manual dexterity, aiming and catching, and balance are also commonly included in task-oriented motor programs. Instead, the intended distinction concerned how these activities were organized and delivered. Relative to a more teacher-directed format, the present program was designed to provide limited but meaningful choices, individually adjusted task difficulty, specific process-focused feedback, recognition of personal improvement, structured opportunities for autonomy, and cooperative peer activities. The need-supportive component therefore concerned the pedagogical and interpersonal conditions surrounding repeated motor practice rather than the motor-practice content itself. However, because the study did not include an active comparison group receiving the same motor content, session frequency, intervention duration, and progression schedule under a conventional teaching format, it could not determine whether need-supportive delivery provided benefits beyond repeated and progressively structured motor practice.

The authors maintained contemporaneous session records for all 30 planned sessions and recorded individual attendance for each participant. This study also documented protocol deviations and intervention-related adverse events throughout the intervention. However, the original study protocol did not include a structured independent observational assessment of specific need-supportive teaching behaviors. Therefore, the available implementation records documented program delivery, participant attendance, and safety but did not provide quantitative component-level fidelity scores for autonomy-supportive, competence-supportive, relatedness-supportive, or structured teaching components.

Phase 1 (Weeks 1–2): Adaptation and supportive learning routines. This phase aimed to familiarize children with the intervention environment, teaching routines, safety rules, and teacher-child relationships. Activities were simple, low-pressure, and success-oriented, including rhythm games, simple object manipulation, basic throwing and catching, and static balance tasks. Teachers emphasized encouragement, clear instructions, and emotional support, and provided simple choices to support autonomy.

Phase 2 (Weeks 3–4): Basic skill acquisition and confidence building. This phase focused on basic manual dexterity, hand-eye coordination, and postural control through structured and progressively graded tasks. Teachers provided demonstrations, step-by-step instructions, and specific feedback. Task difficulty was adjusted based on children’s performance to support repeated success while maintaining an appropriate challenge.

Phase 3 (Weeks 5–6): Skill integration and mid-intervention progression. This phase integrated different motor components within more complex activities, such as catching while stepping, target throwing after balance tasks, obstacle crossing with object transport, and cooperative games. The mid-intervention assessment was conducted after Week 5, and teaching continued with individualized progression and competence-focused feedback.

Phase 4 (Weeks 7–8): Progressive challenge and game-based transfer. This phase increased task complexity and promoted transfer to dynamic game-like contexts. Activities included cooperative target games, relay-style balance challenges, ball control games with changing rules, and multi-step motor sequences. Task constraints such as distance, speed, object size, movement direction, and balance surface were gradually adjusted.

Phase 5 (Weeks 9–10): Consolidation and autonomous application. This phase focused on consolidating previously practiced skills and fostering more independent participation. Children were encouraged to select appropriate task levels, monitor their performance, and indicate which activities they found easier, harder, or more enjoyable. The final week emphasized confidence, participation, and recognition of individual progress, and the post-intervention MABC-2 assessment was conducted after program completion.

### 2.3 Outcome measures

Motor competence was assessed at T0, T1, and T2 using the Movement Assessment Battery for Children-Second Edition (MABC-2), Age Band 2, for children aged 7–10 years. The MABC-2 includes eight motor tasks grouped into three domains: Manual Dexterity, Aiming and Catching, and Balance. Raw task scores were converted into age-adjusted standard scores according to the test manual, with higher scores indicating better motor performance. Wherever possible, the same standardized instructions, scoring procedures, and test sequence were used across the three time points. Trained examiners administered assessments to ensure consistency. The MABC-2 was the only standardized motor outcome measure administered across all three time points. This study did not include untrained motor-transfer tasks or separate standardized measures of physical education participation, play, sport, school-related motor functioning, or daily motor activities.

#### 2.3.1 MABC-2 total and domain standard scores

Outcomes included the MABC-2 total standard score and the Manual Dexterity, Aiming and Catching, and Balance standard scores measured at T0, T1, and T2. The total standard score summarized overall motor competence across the three domains. Manual Dexterity reflected fine motor control and hand manipulation, Aiming and Catching reflected object-control skills and visually guided coordination, and Balance reflected static and dynamic postural control.

#### 2.3.2 Supplementary task-level analyses

Supplementary task-level analyses described changes in individual MABC-2 tasks, including peg placement with the dominant and non-dominant hands, threading beads, drawing trail, catching or beanbag catching, throwing at target, one-leg balance on the preferred and non-preferred legs, walking on tiptoes, and hopping on mats with the preferred and non-preferred legs. These analyses were exploratory and were reported in the supplementary materials to contextualize the total and domain standard score findings.

Implementation-related indicators included the number of planned and delivered sessions, individual participant attendance, participant retention and withdrawal, protocol deviations, and intervention-related adverse events. These indicators were treated as descriptive measures of intervention delivery, participant exposure, retention, and safety rather than as measures of efficacy.

### 2.4 Statistical analysis

All statistical analyses were conducted using R software (Version 4.3.1, R Foundation for Statistical Computing, Vienna, Austria). Analyses assessed within-participant changes in the MABC-2 total and domain standard scores across T0, T1, and T2. Task-specific MABC-2 scores were analyzed separately as supplementary exploratory analyses.

Descriptive statistics were reported as mean ± standard deviation (SD) and median with interquartile range (IQR), as appropriate. Normality of change scores was examined using the Shapiro–Wilk test. Because most change scores deviated from normality, Friedman tests were used to examine performance changes across time points in the MABC-2 total and domain standard scores. When the Friedman test indicated a significant performance change across the time points, Wilcoxon signed-rank tests were conducted for T0 versus T1, T1 versus T2, and T0 versus T2, with Holm adjustment for multiple pairwise comparisons within each outcome. Kendall’s *W* was reported as the effect size for Friedman tests and r for Wilcoxon signed-rank tests.

Task-specific outcomes were analyzed using the same non-parametric approach. A false discovery rate (FDR) adjustment was applied to the omnibus Friedman tests across task-specific outcomes, followed by Holm-adjusted Wilcoxon signed-rank tests where appropriate. Statistical significance was set at *p* < 0.05. Because all participants completed the three MABC-2 assessments, no missing-data imputation was required (Table 1).

**Table 1:** Baseline characteristics of the participants.

Characteristic	Value
Participants, *n*	20
Sex, *n* (%)	
Male	13 (65.0)
Female	7 (35.0)
Age, years	8.38 ± 1.00
Age range, years	7.0–10.0

Values are presented as *n* (%) or mean ± standard deviation unless otherwise indicated.

## 3 Results

### 3.1 Intervention delivery and participant retention

All 20 enrolled participants completed the intervention and all three MABC-2 assessments. All 30 planned intervention sessions were delivered (100%). Participants attended a mean of 29.80 ± 0.52 sessions, corresponding to a mean attendance rate of 99.3%. Seventeen participants attended all 30 sessions, two attended 29 sessions, and one attended 28 sessions. No participants withdrew, and no protocol deviations or intervention-related adverse events were recorded.

### 3.2 MABC-2 total and domain-level outcomes

Table 2 and Figure 2 present the changes in MABC-2 standard scores across T0, T1, and T2, with omnibus and pairwise comparisons reported in Tables 3, 4.

**Table 2:** Descriptive statistics of MABC-2 scores across three time points.

Outcome	Time point	Mean ± SD	Median (Q1, Q3)
MABC-2 total standard score	T0 (pretest)	5.45 ± 1.47	5.50 (5.00, 6.25)
	T1 (midtest)	5.70 ± 1.42	6.00 (5.00, 7.00)
	T2 (posttest)	6.70 ± 1.42	7.00 (6.00, 8.00)
Manual dexterity standard score	T0 (pretest)	4.80 ± 2.24	4.00 (3.75, 5.25)
	T1 (midtest)	4.85 ± 2.06	5.00 (3.75, 6.00)
	T2 (posttest)	5.65 ± 2.06	5.00 (4.00, 7.00)
Aiming and catching standard score	T0 (pretest)	7.55 ± 2.28	8.00 (7.00, 9.00)
	T1 (midtest)	7.85 ± 2.21	8.00 (7.00, 9.00)
	T2 (posttest)	9.65 ± 2.70	10.00 (8.75, 11.25)
Balance standard score	T0 (pretest)	7.30 ± 2.00	8.00 (6.00, 9.00)
	T1 (midtest)	7.50 ± 2.06	8.00 (6.00, 9.00)
	T2 (posttest)	8.15 ± 1.98	9.00 (8.00, 9.00)

Values are presented as mean ± standard deviation and median (first quartile, third quartile). MABC-2, Movement Assessment Battery for Children, Second Edition; SD, standard deviation; Q1, first quartile; Q3, third quartile; T0, pretest, T1, midtest, T2, posttest. All scores are standard scores, and *n* = 20 at each time point.

**Figure 2:**
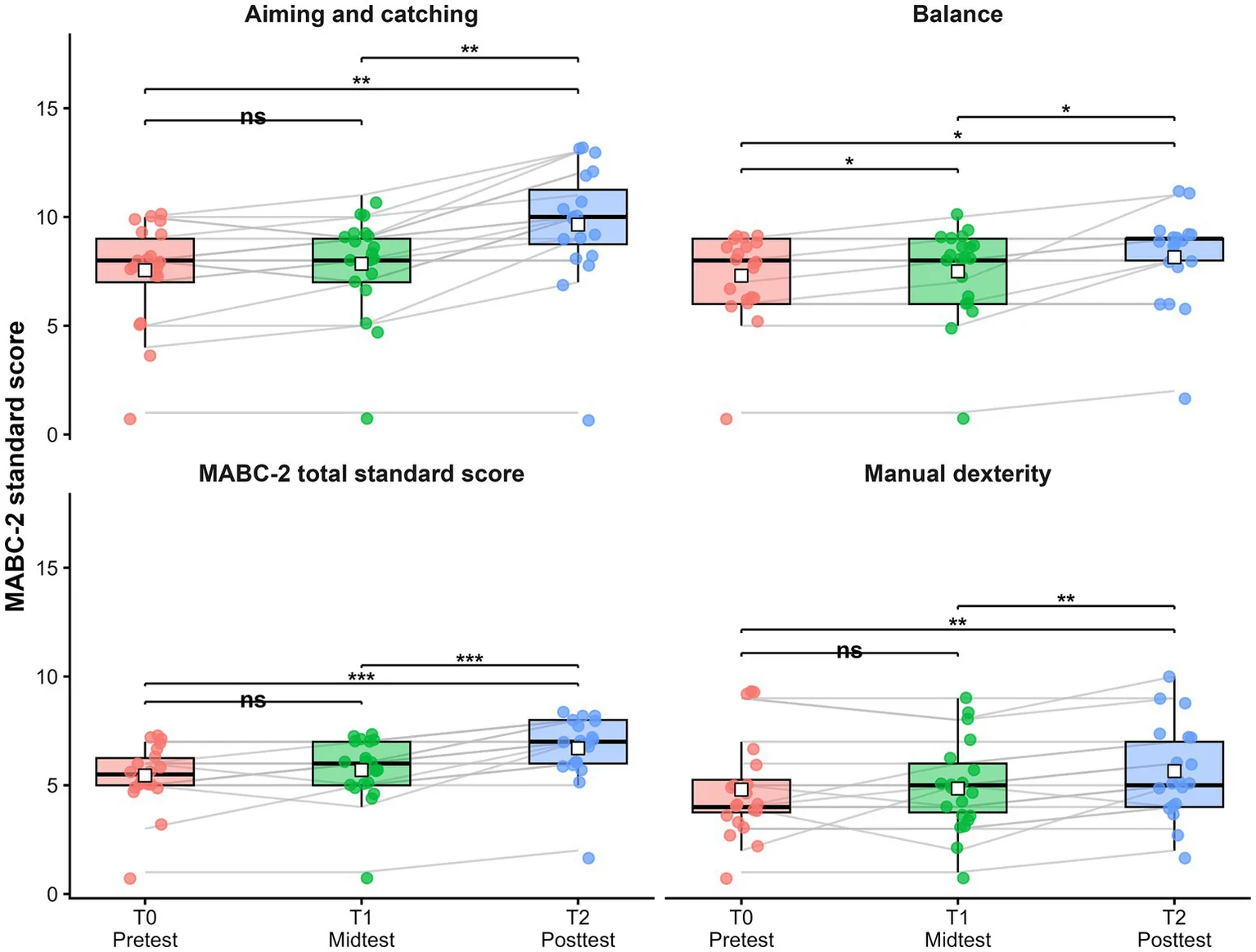
Changes in MABC-2 standard scores across three time points. Grey lines indicate individual trajectories. Boxplots show median and interquartile range, and white squares indicate means. Pairwise comparisons were performed using Wilcoxon signed-rank tests with Holm correction. MABC-2, Movement Assessment Battery for Children, Second Edition; T0, pretest; T1, midtest; T2, posttest. *p < 0.05, **p < 0.01, ***p < 0.001; ns, not significant.

**Table 3:** Results of Friedman tests for MABC-2 standard scores across three time points.

Outcome	Friedman *χ*^2^	df	*p* value	Kendall’s *W*
MABC-2 total standard score	27.825	2	<0.001	0.696
Manual dexterity standard score	20.320	2	<0.001	0.508
Aiming and catching standard score	25.033	2	<0.001	0.626
Balance standard score	14.966	2	<0.001	0.374

Friedman tests were used to compare scores across the three repeated measurement time points (T0, pretest; T1, midtest; T2, posttest) within the same participants. MABC-2, Movement Assessment Battery for Children, Second Edition; df, degrees of freedom. Kendall’s *W* was reported as the effect size. A *p*-value < 0.05 was considered statistically significant.

**Table 4:** *Post hoc* pairwise comparisons of MABC-2 standard scores using Wilcoxon signed-rank tests with Holm correction.

Outcome	Comparison	Mean change ± SD	*Z*	*V*	*p* value	Holm-adjusted *p* value	*r*
MABC-2 total standard score	T0 vs. T1	0.25 ± 0.72	1.507	28.000	0.132	0.132	0.337
MABC-2 total standard score	T1 vs. T2	1.00 ± 0.79	3.573	120.000	<0.001	<0.001	0.799
MABC-2 total standard score	T0 vs. T2	1.25 ± 0.85	3.743	153.000	<0.001	<0.001	0.837
Manual dexterity standard score	T0 vs. T1	0.05 ± 1.05	0.174	15.000	0.863	0.863	0.039
Manual dexterity standard score	T1 vs. T2	0.80 ± 0.83	3.260	113.000	0.001	0.002	0.729
Manual dexterity standard score	T0 vs. T2	0.85 ± 0.81	3.354	91.000	<0.001	0.002	0.750
Aiming and catching standard score	T0 vs. T1	0.30 ± 0.86	1.498	65.000	0.134	0.134	0.335
Aiming and catching standard score	T1 vs. T2	1.80 ± 1.40	3.444	120.000	<0.001	0.001	0.770
Aiming and catching standard score	T0 vs. T2	2.10 ± 1.41	3.573	136.000	<0.001	0.001	0.799
Balance standard score	T0 vs. T1	0.20 ± 0.41	1.999	10.000	0.046	0.046	0.447
Balance standard score	T1 vs. T2	0.65 ± 1.14	2.410	28.000	0.016	0.032	0.539
Balance standard score	T0 vs. T2	0.85 ± 1.31	2.715	45.000	0.007	0.020	0.607

Wilcoxon signed-rank tests were used for post hoc pairwise comparisons after significant Friedman tests. Mean change was calculated as the later time point minus the earlier time point; positive values indicate improvement. Holm-adjusted p-values account for multiple comparisons within each outcome. MABC-2, Movement Assessment Battery for Children, Second Edition; T0, pretest; T1, midtest; T2, posttest; SD, standard deviation; V, Wilcoxon signed-rank statistic; Z, standardized Wilcoxon test statistic; *r*, effect size for Wilcoxon signed-rank tests. A *p*-value < 0.05 was considered statistically significant.

The MABC-2 total standard score increased from 5.45 ± 1.47 at T0 to 5.70 ± 1.42 at T1 and 6.70 ± 1.42 at T2. The Friedman test indicated a significant change across time points for the total standard score, *χ*^2^(2) = 27.825, *p* < 0.001, Kendall’s *W* = 0.696. *Post hoc* Wilcoxon signed-rank tests with Holm adjustment showed no significant difference between T0 and T1 (adjusted *p* = 0.132) but significant differences between T1 and T2 (adjusted *p* < 0.001) and between T0 and T2 (adjusted *p* < 0.001).

Manual Dexterity scores were 4.80 ± 2.24 at T0, 4.85 ± 2.06 at T1, and 5.65 ± 2.06 at T2. The Friedman test showed a significant performance change across the time points, *χ*^2^(2) = 20.320, *p* < 0.001, Kendall’s *W* = 0.508. Holm-adjusted Wilcoxon signed-rank tests showed no significant difference between T0 and T1 (adjusted *p* = 0.863) but significant differences between T1 and T2 (adjusted *p* = 0.002) and between T0 and T2 (adjusted *p* = 0.002).

Aiming and Catching scores were 7.55 ± 2.28 at T0, 7.85 ± 2.21 at T1, and 9.65 ± 2.70 at T2. The Friedman test showed a significant performance change across the time points, χ^2^(2) = 25.033, *p* < 0.001, Kendall’s *W* = 0.626. Holm-adjusted Wilcoxon signed-rank tests showed no significant difference between T0 and T1 (adjusted *p* = 0.134), but significant differences between T1 and T2 (adjusted*χ*^2^ *p* = 0.001) and between T0 and T2 (adjusted *p* = 0.001).

Balance scores were 7.30 ± 2.00 at T0, 7.50 ± 2.06 at T1, and 8.15 ± 1.98 at T2. The Friedman test indicated a significant change in performance across the time points, *χ*^2^(2) = 14.966, *p* < 0.001, Kendall’s *W* = 0.374. Holm-adjusted Wilcoxon signed-rank tests showed significant differences between T0 and T1 (adjusted *p* = 0.046), T1 and T2 (adjusted *p* = 0.032), and T0 and T2 (adjusted *p* = 0.020).

### 3.3 Exploratory task-specific outcomes

Exploratory task-specific MABC-2 results are reported in the supplementary materials. Supplementary Table S2 presents descriptive statistics for individual MABC-2 tasks at T0, T1, and T2, while Supplementary Tables S3, S4 report the corresponding pairwise comparisons. Significant corrected task-level changes were observed for peg placement with both the dominant and non-dominant hands, threading beads, catching/beanbag catching, throwing at target, selected one-leg balance tasks, and hopping on mats with the non-preferred leg. Drawing trail, walking on tiptoes, and hopping on mats with the preferred leg did not show significant corrected changes across the relevant comparisons. These task-specific analyses were exploratory and supplementary to the MABC-2 total and domain standard score outcomes.

## 4 Discussion

This single-group repeated-measures study examined changes in motor competence during a 10-week need-supportive teaching intervention among children aged 7–10 years with motor difficulties identified through school-based screening. The findings showed within-participant improvements in overall motor competence, with significant differences emerging after the intervention period rather than during the first 4 weeks. Improvements were observed across all three MABC-2 domains, though the magnitude and timing of changes varied by domain. These findings suggest that a school-based need-supportive teaching approach may provide a feasible context for supporting motor practice and skill development in children with motor difficulties. However, because the study used a single-group design without a comparison condition, the observed changes should be interpreted as within-participant changes over time rather than definitive evidence of intervention effectiveness.

The different patterns of change across MABC-2 domains may reflect differences in task demands, movement characteristics, and opportunities for practice. Aiming and Catching showed the largest improvement, which may partly reflect the frequent inclusion of throwing, catching, and target-oriented activities during the intervention. These activities provided repeated opportunities for goal-directed practice, immediate feedback, and adjustment of movement strategies. This interpretation aligns with evidence that task-oriented and activity-oriented interventions can promote motor improvements when practice conditions closely match functional movement demands (Smits-Engelsman et al., 2013). Manual Dexterity also improved, although changes appeared later than those observed for object-control skills. Fine motor tasks require precise control, coordination, and repeated practice opportunities, which may contribute to slower observable changes. Balance showed smaller but significant improvements, potentially reflecting the complex interaction between postural control, environmental constraints, and task-specific balance demands (Jelsma et al., 2026).

The present findings should also be considered in light of recent DCD intervention literature. Recent reviews have highlighted that effective motor interventions for children with DCD or motor difficulties typically involve structured practice, meaningful task engagement, and sufficient opportunities for repetition rather than isolated skill training alone (Gao et al., 2025; Jelsma et al., 2026). Similarly, recent research has emphasized the importance of accounting for task specificity and individual variability when interpreting changes in motor performance. For example, Shahbaz et al. (2024) reported that motivationally supportive practice conditions combined with self-controlled practice could influence motor learning outcomes in children identified by the original study as being at risk for DCD. However, effects varied across practice, retention, and transfer conditions. Together with previous evidence on motor-based and activity-oriented interventions (Smits-Engelsman et al., 2013; Purpura et al., 2024), these findings suggest that integrating supportive pedagogical strategies into motor practice may represent a promising approach for school-based intervention contexts.

From a theoretical perspective, need-supportive teaching provides a potential framework for understanding how the intervention environment may have supported motor learning. The use of meaningful choices, progressively graded challenges, specific process-focused feedback, and cooperative activities reflects principles consistent with autonomy support, competence support, and relatedness support within self-determination theory (Ryan and Deci, 2000). Previous research in physical education has shown that need-supportive teaching approaches are associated with more adaptive motivational experiences, greater psychological need satisfaction, and increased engagement (Mouratidis et al., 2008; Behzadnia et al., 2022). However, the present study did not directly assess children’s perceived autonomy support, psychological need satisfaction, motivation, or engagement. Therefore, these mechanisms should be considered as plausible interpretations rather than confirmed explanations for the observed motor changes. Future studies should incorporate psychological and motivational measures to clarify whether changes in these processes contribute to motor development.

The intervention also demonstrated practical feasibility within a school-based setting. All planned sessions were delivered, participant attendance was high, and no intervention-related adverse events or protocol deviations were recorded. These findings suggest that need-supportive teaching principles can be incorporated into school-based motor activities while maintaining high participation. This approach is feasible because children with motor difficulties often experience reduced participation, avoidance, and negative movement-related experiences, which may limit opportunities for continued motor practice (Zimmer et al., 2020). Nevertheless, intervention fidelity was monitored through session records rather than independent observation-based assessment, and the present study did not include a manipulation check to determine whether children actually perceived greater autonomy support, competence support, or relatedness support.

## 5 Limitations

Several limitations should be considered when interpreting the present findings. First, the single-group repeated-measures design limits causal interpretation because changes observed across T0, T1, and T2 cannot be definitively attributed to the intervention alone. The absence of a comparison group means that maturation, repeated testing effects, or other contextual factors cannot be fully ruled out. Future randomized controlled studies with larger samples and appropriate comparison conditions are needed to determine the effectiveness of need-supportive teaching interventions for improving motor competence.

Second, interpret participant identification cautiously. Participants were identified as having motor difficulties based on questionnaire screening and MABC-2 performance at or below the 16th percentile; however, the authors did not conduct a comprehensive clinical diagnostic assessment for DCD. Standardized diagnostic procedures were not used to assess functional impairment in daily living or school participation and co-occurring neurodevelopmental conditions. Therefore, the present sample should not be considered equivalent to children with clinically confirmed DCD, and the findings should be interpreted within the context of school-based identification of motor difficulties rather than clinical diagnosis.

Third, intervention fidelity and psychological mechanisms were not independently assessed. Although session records documented attendance, intervention completion, protocol deviations, and adverse events, the authors did not conduct an external observation-based fidelity assessment or manipulation check to verify the extent to which need-supportive components were consistently delivered or perceived by participants. Furthermore, changes in psychological processes such as autonomy support, psychological need satisfaction, motivation, and engagement were not measured. Future research should incorporate objective fidelity assessments and mechanism-related outcomes to clarify how need-supportive teaching influences motor development.

Finally, some intervention activities overlapped with MABC-2 task demands, which may have contributed to improvements through task-specific practice or familiarity with similar movement requirements. Although this overlap reflects ecological alignment between training and assessment, it limits conclusions regarding broader transfer of motor competence beyond the measured tasks. Furthermore, structured learning routines, graded motor tasks, and repeated practice opportunities were delivered as integrated components of the intervention package and were not independently manipulated. Therefore, the specific contribution of need-supportive teaching principles relative to other intervention components cannot be determined. Future studies should include transfer measures, longer-term follow-up, and designs that can isolate the effects of specific intervention components.

## 6 Conclusion

Children aged 7–10 years with motor difficulties showed positive within-participant changes in MABC-2 total and domain standard scores during the 10-week need-supportive teaching program. Improvements were most evident from T1 to T2, and Aiming and Catching showed the largest mean increase from baseline to post-intervention. These findings support the potential value of integrating need-supportive teaching principles with structured motor practice in school-based settings. However, the results should be interpreted cautiously because of the single-group design, small sample size, and possible task-specific practice effects.

## Data Availability

The original contributions presented in the study are included in the article/Supplementary material, further inquiries can be directed to the corresponding authors.
