# Supplementary material for: Effects of a 10-week need-supportive teaching program on motor competence in children ages 7–10 with motor difficulties: a single-group repeated-measures design
Source: Front Psychol. 2026 Sep 24;17:1909241. doi: 10.3389/fpsyg.2026.1909241 (PMC13645559; doi:10.3389/fpsyg.2026.1909241)
Supplement: Supplementary file 1 [file Table_1.DOCX]

**Table S1. Normality tests for change scores of MABC-2 standard scores**

| **Outcome** | **Change score** | **n** | **Mean ± SD** | **Median** | **Range** | **W** | **p-value** | **Interpretation** |
| --- | --- | --- | --- | --- | --- | --- | --- | --- |
| MABC-2 total standard score | T1 - T0 | 20 | 0.25 ± 0.72 | 0.00 | -1 to 2 | 0.821 | 0.002 | Non-normal |
| MABC-2 total standard score | T2 - T1 | 20 | 1.00 ± 0.79 | 1.00 | 0 to 3 | 0.832 | 0.003 | Non-normal |
| MABC-2 total standard score | T2 - T0 | 20 | 1.25 ± 0.85 | 1.00 | 0 to 3 | 0.844 | 0.004 | Non-normal |
| Manual dexterity standard score | T1 - T0 | 20 | 0.05 ± 1.05 | 0.00 | -2 to 3 | 0.784 | <0.001 | Non-normal |
| Manual dexterity standard score | T2 - T1 | 20 | 0.80 ± 0.83 | 1.00 | -1 to 3 | 0.827 | 0.002 | Non-normal |
| Manual dexterity standard score | T2 - T0 | 20 | 0.85 ± 0.81 | 1.00 | 0 to 3 | 0.809 | 0.001 | Non-normal |
| Aiming and catching standard score | T1 - T0 | 20 | 0.30 ± 0.86 | 0.00 | -1 to 2 | 0.867 | 0.010 | Non-normal |
| Aiming and catching standard score | T2 - T1 | 20 | 1.80 ± 1.40 | 2.00 | 0 to 4 | 0.884 | 0.021 | Non-normal |
| Aiming and catching standard score | T2 - T0 | 20 | 2.10 ± 1.41 | 2.00 | 0 to 5 | 0.913 | 0.072 | Normal |
| Balance standard score | T1 - T0 | 20 | 0.20 ± 0.41 | 0.00 | 0 to 1 | 0.495 | <0.001 | Non-normal |
| Balance standard score | T2 - T1 | 20 | 0.65 ± 1.14 | 0.00 | 0 to 4 | 0.646 | <0.001 | Non-normal |
| Balance standard score | T2 - T0 | 20 | 0.85 ± 1.31 | 0.00 | 0 to 5 | 0.701 | <0.001 | Non-normal |

*Note.* Values are change scores calculated as T1 - T0, T2 - T1, and T2 - T0. Normality was assessed using the Shapiro-Wilk test. A p-value < 0.05 indicates deviation from normality. Non-normal = not normally distributed. MABC-2 = Movement Assessment Battery for Children, Second Edition; SD = standard deviation; T0 = pretest; T1 = midtest; T2 = posttest.

**Table S2. Descriptive statistics of task-specific MABC-2 scores across three time points**

| **Task-specific score** | **Time point** | **n** | **Mean ± SD** | **Median (Q1, Q3)** |
| --- | --- | --- | --- | --- |
| Placing pegs, dominant hand | T0 (pretest) | 20 | 5.95 ± 5.01 | 5.00 (1.00, 10.25) |
| Placing pegs, dominant hand | T1 (midtest) | 20 | 6.70 ± 4.50 | 6.50 (2.75, 10.25) |
| Placing pegs, dominant hand | T2 (posttest) | 20 | 8.05 ± 3.93 | 8.00 (5.75, 11.00) |
| Placing pegs, non-dominant hand | T0 (pretest) | 20 | 6.15 ± 5.02 | 6.00 (1.00, 9.50) |
| Placing pegs, non-dominant hand | T1 (midtest) | 20 | 6.75 ± 4.54 | 7.00 (2.75, 9.50) |
| Placing pegs, non-dominant hand | T2 (posttest) | 20 | 7.65 ± 4.04 | 7.00 (4.75, 10.25) |
| Threading beads | T0 (pretest) | 20 | 6.55 ± 2.39 | 7.00 (5.00, 8.00) |
| Threading beads | T1 (midtest) | 20 | 6.70 ± 2.52 | 7.00 (6.00, 8.00) |
| Threading beads | T2 (posttest) | 20 | 7.80 ± 2.59 | 8.00 (7.00, 9.00) |
| Drawing trail | T0 (pretest) | 20 | 2.50 ± 2.33 | 1.00 (1.00, 3.50) |
| Drawing trail | T1 (midtest) | 20 | 3.25 ± 3.26 | 1.50 (1.00, 4.25) |
| Drawing trail | T2 (posttest) | 20 | 3.15 ± 2.98 | 2.00 (1.00, 4.25) |
| Catching/beanbag catching | T0 (pretest) | 20 | 7.85 ± 2.21 | 8.00 (7.00, 9.00) |
| Catching/beanbag catching | T1 (midtest) | 20 | 8.60 ± 2.21 | 9.00 (8.00, 10.00) |
| Catching/beanbag catching | T2 (posttest) | 20 | 9.45 ± 2.67 | 10.00 (8.00, 11.00) |
| Throwing at target | T0 (pretest) | 20 | 6.80 ± 2.31 | 6.00 (5.75, 8.00) |
| Throwing at target | T1 (midtest) | 20 | 7.75 ± 2.92 | 7.00 (6.00, 9.00) |
| Throwing at target | T2 (posttest) | 20 | 8.35 ± 2.23 | 8.50 (7.50, 9.25) |
| One-leg balance, preferred leg | T0 (pretest) | 20 | 5.75 ± 1.12 | 6.00 (5.00, 6.00) |
| One-leg balance, preferred leg | T1 (midtest) | 20 | 5.20 ± 1.70 | 6.00 (5.00, 6.00) |
| One-leg balance, preferred leg | T2 (posttest) | 20 | 6.30 ± 1.87 | 6.00 (6.00, 7.00) |
| One-leg balance, non-preferred leg | T0 (pretest) | 20 | 7.10 ± 0.85 | 7.00 (7.00, 8.00) |
| One-leg balance, non-preferred leg | T1 (midtest) | 20 | 6.80 ± 1.32 | 7.00 (7.00, 7.00) |
| One-leg balance, non-preferred leg | T2 (posttest) | 20 | 7.65 ± 1.39 | 7.00 (7.00, 8.00) |
| Walking on tiptoes | T0 (pretest) | 20 | 8.05 ± 3.85 | 9.50 (7.75, 11.00) |
| Walking on tiptoes | T1 (midtest) | 20 | 8.20 ± 3.52 | 9.00 (7.75, 11.00) |
| Walking on tiptoes | T2 (posttest) | 20 | 9.05 ± 2.37 | 9.50 (8.00, 11.00) |
| Hopping on mats, preferred leg | T0 (pretest) | 20 | 9.95 ± 2.56 | 11.00 (10.75, 11.00) |
| Hopping on mats, preferred leg | T1 (midtest) | 20 | 9.95 ± 2.58 | 11.00 (11.00, 11.00) |
| Hopping on mats, preferred leg | T2 (posttest) | 20 | 10.05 ± 2.61 | 11.00 (11.00, 11.00) |
| Hopping on mats, non-preferred leg | T0 (pretest) | 20 | 9.35 ± 3.10 | 10.50 (8.75, 12.00) |
| Hopping on mats, non-preferred leg | T1 (midtest) | 20 | 9.25 ± 3.01 | 10.00 (9.00, 11.25) |
| Hopping on mats, non-preferred leg | T2 (posttest) | 20 | 10.05 ± 2.87 | 11.00 (9.75, 12.00) |

**Note.** Values are presented as mean ± standard deviation and median (first quartile, third quartile). MABC-2 = Movement Assessment Battery for Children, Second Edition; SD = standard deviation; Q1 = first quartile; Q3 = third quartile; T0 = pretest; T1 = midtest; T2 = posttest.

**Table S3. Post hoc pairwise comparisons of all task-specific MABC-2 scores using Wilcoxon signed-rank tests with Holm correction**

| **Task-specific score** | **Comparison** | **n** | **Mean change ± SD** | **Median change** | **V** | **p value** | **Holm-adjusted p value** | **r** | **Sig.** |
| --- | --- | --- | --- | --- | --- | --- | --- | --- | --- |
| Placing pegs, dominant hand | T0 vs T1 | 20 | 0.75 ± 1.29 | 0.00 | 36.000 | 0.01 | 0.015 | 0.578 | * |
| Placing pegs, dominant hand | T1 vs T2 | 20 | 1.35 ± 1.90 | 0.00 | 45.000 | 0.008 | 0.015 | 0.598 | * |
| Placing pegs, dominant hand | T0 vs T2 | 20 | 2.10 ± 2.63 | 1.00 | 66.000 | 0.003 | 0.009 | 0.660 | ** |
| Placing pegs, non-dominant hand | T0 vs T1 | 20 | 0.60 ± 1.14 | 0.00 | 41.000 | 0.021 | 0.042 | 0.516 | * |
| Placing pegs, non-dominant hand | T1 vs T2 | 20 | 0.90 ± 1.86 | 0.00 | 64.500 | 0.039 | 0.042 | 0.462 | * |
| Placing pegs, non-dominant hand | T0 vs T2 | 20 | 1.50 ± 2.16 | 1.00 | 72.500 | 0.008 | 0.025 | 0.591 | * |
| Threading beads | T0 vs T1 | 20 | 0.15 ± 1.27 | 0.00 | 38.500 | 0.617 | 0.617 | 0.112 | ns |
| Threading beads | T1 vs T2 | 20 | 1.10 ± 1.33 | 1.00 | 78.000 | 0.002 | 0.005 | 0.700 | ** |
| Threading beads | T0 vs T2 | 20 | 1.25 ± 2.17 | 0.00 | 51.000 | 0.016 | 0.032 | 0.539 | * |
| Drawing trail | T0 vs T1 | 20 | 0.75 ± 2.27 | 0.00 | 17.000 | 0.168 | 0.505 | 0.308 | ns |
| Drawing trail | T1 vs T2 | 20 | -0.10 ± 2.65 | 0.00 | 26.500 | 0.632 | 0.632 | 0.107 | ns |
| Drawing trail | T0 vs T2 | 20 | 0.65 ± 2.23 | 0.00 | 16.000 | 0.248 | 0.505 | 0.259 | ns |
| Catching/beanbag catching | T0 vs T1 | 20 | 0.75 ± 1.12 | 1.00 | 93.000 | 0.007 | 0.014 | 0.602 | * |
| Catching/beanbag catching | T1 vs T2 | 20 | 0.85 ± 1.18 | 0.50 | 62.500 | 0.007 | 0.014 | 0.598 | * |
| Catching/beanbag catching | T0 vs T2 | 20 | 1.60 ± 1.64 | 2.00 | 89.500 | 0.002 | 0.006 | 0.693 | ** |
| Throwing at target | T0 vs T1 | 20 | 0.95 ± 1.50 | 1.00 | 107.000 | 0.005 | 0.014 | 0.635 | * |
| Throwing at target | T1 vs T2 | 20 | 0.60 ± 2.62 | 1.00 | 76.500 | 0.128 | 0.128 | 0.341 | ns |
| Throwing at target | T0 vs T2 | 20 | 1.55 ± 1.93 | 2.00 | 92.000 | 0.012 | 0.024 | 0.561 | * |
| One-leg balance, preferred leg | T0 vs T1 | 20 | -0.55 ± 1.05 | 0.00 | 3.000 | 0.031 | 0.062 | 0.482 | ns |
| One-leg balance, preferred leg | T1 vs T2 | 20 | 1.10 ± 1.41 | 1.00 | 66.000 | 0.003 | 0.008 | 0.669 | ** |
| One-leg balance, preferred leg | T0 vs T2 | 20 | 0.55 ± 1.23 | 0.00 | 32.500 | 0.034 | 0.062 | 0.475 | ns |
| One-leg balance, non-preferred leg | T0 vs T1 | 20 | -0.30 ± 1.08 | 0.00 | 5.000 | 0.236 | 0.236 | 0.265 | ns |
| One-leg balance, non-preferred leg | T1 vs T2 | 20 | 0.85 ± 1.14 | 0.50 | 55.000 | 0.004 | 0.012 | 0.642 | * |
| One-leg balance, non-preferred leg | T0 vs T2 | 20 | 0.55 ± 1.15 | 0.00 | 47.000 | 0.039 | 0.078 | 0.462 | ns |
| Walking on tiptoes | T0 vs T1 | 20 | 0.15 ± 0.81 | 0.00 | 10.000 | 0.48 | 0.48 | 0.158 | ns |
| Walking on tiptoes | T1 vs T2 | 20 | 0.85 ± 1.69 | 0.00 | 48.000 | 0.034 | 0.084 | 0.475 | ns |
| Walking on tiptoes | T0 vs T2 | 20 | 1.00 ± 2.08 | 0.00 | 33.500 | 0.028 | 0.084 | 0.491 | ns |
| Hopping on mats, preferred leg | T0 vs T1 | 20 | 0.00 ± 0.32 | 0.00 | 1.500 | 1.0 | 1.0 | 0.000 | ns |
| Hopping on mats, preferred leg | T1 vs T2 | 20 | 0.10 ± 0.31 | 0.00 | 3.000 | 0.157 | 0.472 | 0.316 | ns |
| Hopping on mats, preferred leg | T0 vs T2 | 20 | 0.10 ± 0.45 | 0.00 | 1.000 | 0.317 | 0.635 | 0.224 | ns |
| Hopping on mats, non-preferred leg | T0 vs T1 | 20 | -0.10 ± 0.64 | 0.00 | 5.000 | 0.48 | 0.48 | 0.158 | ns |
| Hopping on mats, non-preferred leg | T1 vs T2 | 20 | 0.80 ± 0.95 | 0.50 | 55.000 | 0.004 | 0.013 | 0.639 | * |
| Hopping on mats, non-preferred leg | T0 vs T2 | 20 | 0.70 ± 1.08 | 0.00 | 28.000 | 0.017 | 0.033 | 0.535 | * |

**Note.** Wilcoxon signed-rank tests were used for post hoc pairwise comparisons. Mean change was calculated as the later time point minus the earlier time point; positive values indicate a higher recorded task-specific score at the later time point. Holm-adjusted p values account for multiple comparisons within each task-specific outcome. V = Wilcoxon signed-rank statistic; r = effect size for Wilcoxon signed-rank tests; Sig. = significance based on Holm-adjusted p values. * p < 0.05, ** p < 0.01, *** p < 0.001; ns = not significant.

**Table S4. Post hoc pairwise comparisons for task-specific MABC-2 scores with significant FDR-adjusted Friedman tests**

| **Task-specific score** | **Comparison** | **n** | **Mean change ± SD** | **Median change** | **V** | **p value** | **Holm-adjusted p value** | **r** | **Sig.** |
| --- | --- | --- | --- | --- | --- | --- | --- | --- | --- |
| Placing pegs, dominant hand | T0 vs T1 | 20 | 0.75 ± 1.29 | 0.00 | 36.000 | 0.01 | 0.015 | 0.578 | * |
| Placing pegs, dominant hand | T1 vs T2 | 20 | 1.35 ± 1.90 | 0.00 | 45.000 | 0.008 | 0.015 | 0.598 | * |
| Placing pegs, dominant hand | T0 vs T2 | 20 | 2.10 ± 2.63 | 1.00 | 66.000 | 0.003 | 0.009 | 0.660 | ** |
| Placing pegs, non-dominant hand | T0 vs T1 | 20 | 0.60 ± 1.14 | 0.00 | 41.000 | 0.021 | 0.042 | 0.516 | * |
| Placing pegs, non-dominant hand | T1 vs T2 | 20 | 0.90 ± 1.86 | 0.00 | 64.500 | 0.039 | 0.042 | 0.462 | * |
| Placing pegs, non-dominant hand | T0 vs T2 | 20 | 1.50 ± 2.16 | 1.00 | 72.500 | 0.008 | 0.025 | 0.591 | * |
| Threading beads | T0 vs T1 | 20 | 0.15 ± 1.27 | 0.00 | 38.500 | 0.617 | 0.617 | 0.112 | ns |
| Threading beads | T1 vs T2 | 20 | 1.10 ± 1.33 | 1.00 | 78.000 | 0.002 | 0.005 | 0.700 | ** |
| Threading beads | T0 vs T2 | 20 | 1.25 ± 2.17 | 0.00 | 51.000 | 0.016 | 0.032 | 0.539 | * |
| Catching/beanbag catching | T0 vs T1 | 20 | 0.75 ± 1.12 | 1.00 | 93.000 | 0.007 | 0.014 | 0.602 | * |
| Catching/beanbag catching | T1 vs T2 | 20 | 0.85 ± 1.18 | 0.50 | 62.500 | 0.007 | 0.014 | 0.598 | * |
| Catching/beanbag catching | T0 vs T2 | 20 | 1.60 ± 1.64 | 2.00 | 89.500 | 0.002 | 0.006 | 0.693 | ** |
| Throwing at target | T0 vs T1 | 20 | 0.95 ± 1.50 | 1.00 | 107.000 | 0.005 | 0.014 | 0.635 | * |
| Throwing at target | T1 vs T2 | 20 | 0.60 ± 2.62 | 1.00 | 76.500 | 0.128 | 0.128 | 0.341 | ns |
| Throwing at target | T0 vs T2 | 20 | 1.55 ± 1.93 | 2.00 | 92.000 | 0.012 | 0.024 | 0.561 | * |
| One-leg balance, preferred leg | T0 vs T1 | 20 | -0.55 ± 1.05 | 0.00 | 3.000 | 0.031 | 0.062 | 0.482 | ns |
| One-leg balance, preferred leg | T1 vs T2 | 20 | 1.10 ± 1.41 | 1.00 | 66.000 | 0.003 | 0.008 | 0.669 | ** |
| One-leg balance, preferred leg | T0 vs T2 | 20 | 0.55 ± 1.23 | 0.00 | 32.500 | 0.034 | 0.062 | 0.475 | ns |
| One-leg balance, non-preferred leg | T0 vs T1 | 20 | -0.30 ± 1.08 | 0.00 | 5.000 | 0.236 | 0.236 | 0.265 | ns |
| One-leg balance, non-preferred leg | T1 vs T2 | 20 | 0.85 ± 1.14 | 0.50 | 55.000 | 0.004 | 0.012 | 0.642 | * |
| One-leg balance, non-preferred leg | T0 vs T2 | 20 | 0.55 ± 1.15 | 0.00 | 47.000 | 0.039 | 0.078 | 0.462 | ns |
| Walking on tiptoes | T0 vs T1 | 20 | 0.15 ± 0.81 | 0.00 | 10.000 | 0.48 | 0.48 | 0.158 | ns |
| Walking on tiptoes | T1 vs T2 | 20 | 0.85 ± 1.69 | 0.00 | 48.000 | 0.034 | 0.084 | 0.475 | ns |
| Walking on tiptoes | T0 vs T2 | 20 | 1.00 ± 2.08 | 0.00 | 33.500 | 0.028 | 0.084 | 0.491 | ns |
| Hopping on mats, non-preferred leg | T0 vs T1 | 20 | -0.10 ± 0.64 | 0.00 | 5.000 | 0.48 | 0.48 | 0.158 | ns |
| Hopping on mats, non-preferred leg | T1 vs T2 | 20 | 0.80 ± 0.95 | 0.50 | 55.000 | 0.004 | 0.013 | 0.639 | * |
| Hopping on mats, non-preferred leg | T0 vs T2 | 20 | 0.70 ± 1.08 | 0.00 | 28.000 | 0.017 | 0.033 | 0.535 | * |

**Note.** This table includes only task-specific outcomes with significant FDR-adjusted Friedman tests in Table 5. Pairwise comparisons were conducted using Wilcoxon signed-rank tests with Holm correction within each task-specific outcome. Mean change was calculated as the later time point minus the earlier time point. FDR = false discovery rate; MABC-2 = Movement Assessment Battery for Children, Second Edition; SD = standard deviation; V = Wilcoxon signed-rank statistic; r = effect size. * p < 0.05, ** p < 0.01, *** p < 0.001; ns = not significant.
